# Mycobacterial species and their contribution to cholesterol degradation in wastewater treatment plants

**DOI:** 10.1038/s41598-018-37332-w

**Published:** 2019-01-29

**Authors:** Feng Guo, Tong Zhang, Bing Li, Zhiping Wang, Feng Ju, Yi-ting Liang

**Affiliations:** 1 School of Life Sciences, Xiamen University, Xiamen, China; 2 Environmental Biotechnology Laboratory, The University of Hong Kong, Hong Kong SAR, China; 3 Key Laboratory of Microorganism Application and Risk Control of Shenzhen, Graduate School at Shenzhen, Tsinghua University, Shenzhen, China; 4 School of Environmental Science and Engineering, Shanghai Jiao Tong University, Shanghai, China

## Abstract

*Mycobacterium* often presents as an abundant bacterial genus in activated sludge in many wastewater treatment plants (WWTPs), but the species-level taxonomy and functions remain poorly understood. In this study, we profiled the mycobacterial communities in eleven WWTPs from five countries by pyrosequencing the *rpoB* amplicons and searching against a customized database of mycobacterial *rpoB* sequences. Results indicated that major mycobacterial species were related to *M. brumae*, *M. crocinum*, *M. sphagni*, etc., most of which belong to poorly characterized rapidly-growing group. A few opportunistic pathogenic species were detected, suggesting the potential risk of mycobacteria in WWTPs. Genomic analysis of four isolates from activated sludge indicated these genomes contained genes of degradations of alkane, aromatics, steroids and a variety of cytochrome P450 families. Additionally, a few key genes responsible for cholesterol degradation were detected in a full-scale activated sludge metatranscriptomic dataset reported previously and taxonomically assigned to mycobacteria. Evidence showed that all isolates can degrade cholesterol, a major composition of sewage. Relative abundance of mycobacteria in activated sludge was enriched by 4.7 folds after adding cholesterol into the influent for one week. Our results provided the insights into mycobacterial species and functions in WWTPs.

## Introduction

*Mycobacterium* is one of the most significant disease-causing and environmental bacterial genera ubiquitously distributing in aqueous environments^1–4^. For biological wastewater treatment, *Mycobacterium* has been branded as one of the foaming bacteria in activated sludge (AS) due to its high hydrophobic cell surface and enrichment in the foam^5^. However, very few mycobacterial isolates have been obtained from the wastewater treatment plants (WWTPs). Recently, the high throughput sequencing techniques revealed that this genus frequently was present at high abundance in the global WWTPs, especially in municipal WWTPs^6,7^. *Mycobacterium* species in AS accounted for 0.2% to 4% in terms of the 16S rRNA gene abundance, ranging the 2nd to 60th among all classified bacterial genera^7^. Moreover, their relative abundances in terms of cell number could be even higher considering the low copy number (typically 1 or 2 copies) of the rRNA operon in their genomes^8^. These preliminary observations raise the significance to study which *Mycobacterium* species dominate and what are their roles in the AS treatment. On the one hand, the occurrence of pathogenic mycobacteria in WWTPs has not been systematically studied although some case reports were occasionally presented^9^. On the other hand, resolving their roles will help us to further understand the mycobacterial functions in aspects of biotechnology in WWTPs.

Besides of its pathogenic role like causing tuberculosis, lung disease, ulcer, etc., *Mycobacterium* is of environmental significance due to degradation capabilities for aromatic hydrocarbons^10,11^, nitrogen-containing heterocycles, such as morpholines^12^, etc. For example, so far mycobacterial species are the solely reported degraders for morpholine, an important industrial intermediate of wide application^13^. In addition, like some other *Actinobacteria*, many *Mycobacterium* spp. are capable of degrading or transforming steroids^14–16^, which are the typical human fecal components. The sterols, mostly in the form of derivatives of cholesterol, could be as high as several milligrams per gram dry weight level in insoluble form in sewage^17,18^, and are considered as important pollutants due to their estrogenic effects on aquatic invertebrates^19^.

Numerous studies focused on the species or strains identification of the clinical mycobacterial isolates^20–22^. Many species share the very high similar 16S rRNA gene sequences similarities but the low genomic DNA hybridization ratios that indicated that they were distinct organisms^23,24^. So far, partial DNA sequences of rpoB (the β subunit of RNA polymerase) and hsp65 (the 65 kD heat-shock protein) have been proven to be robust methods for species-level mycobacterial identification of isolates and environmental samples^25,26^.

In this study, we aimed to characterize the major mycobacterial species and their potential functions in WWTPs. Mycobacterial species, including potential pathogens, were investigated based on profiling the *rpo*B sequences. Moreover, genomes from four isolates were obtained to get the characteristics of their biodegradation potentials. A major role of mycobacteria for degradation of cholesterol in WWTPs was proposed based on genome annotation, evidence from a full-scale AS metatranscriptomic dataset published previously and further validation by degradation tests with the isolates and enriching mycobacteria in AS by adding cholesterol.

## Methods

### Samples preparation and mycobacterial isolation

The AS samples were collected from the aeration tanks of 11 WWTPs located in five countries. The influents and effluents of two WWTPs from Hong Kong (China) were simultaneously sampled. The detailed information including the relative abundance of *Mycobacterium* for the 16 samples (including a technical duplication for CN-BJ AS sample) was summarized in Table S1. The AS samples were fixed by 50% ethanol (v/v, final concentration) onsite, while the biomass in fresh influent and effluent (transported to lab within 2 h without fixation) were collected by filtering through 0.2 μm pore-sized membranes in lab (100 mL for influent and 1,000 mL for effluent). Then the samples were stored at the −20 °C until DNA extraction.

For the mycobacterial isolation, fresh AS samples from Shatin (ST) and Shekwuhui (SWH) WWTP in Hong Kong were transported to the lab within 2 h. The AS was homogenized manually by a glass tissue homogenizer, and the resulted suspension was serial diluted with 0.85% NaCl by 10^4^ to 10^6^ folds. The mycobacterial selective agar (Middlebrook 7H-11, BD) adding Middlebrook OADC Enrichment (BD) was applied to selectively cultivate *Mycobacterium*. The plates were incubated in 28 °C for 20 days until no new colonies appeared. Colonies were picked according to their shape, size and color and further purified by sequential plate streaking. Their full-length 16S rRNA genes were PCR amplified by using the universal bacterial primer set of 27F and 1492R and sequenced to identify these organisms. Six mycobacterial isolates (one from ST, named ST-F2 and five from SWH named SWH-M1 to SWH-M5) were obtained. Therein, four isolates with >2% 16S rRNA inter-strain divergences were sequenced to retrieve their draft genomes and were deposited in Marine Culture Collection of China with deposit numbers from MCCC 1K02477 to 1K02480.

### DNA extraction, PCR and pyrosequencing of *rpoB*

The fixed AS samples were washed by 0.85% NaCl twice (by centrifugation at 10,000 g for 5 min) and the samples on membrane were detached by vortexing for 1 min after adding 0.85% NaCl and 1-mm acid-washed glass beads. After collecting the biomass by centrifugation, DNA (including AS, biomass on membranes and isolates) was extracted by using the FastDNA SPIN kit for Soil as described before^27^. All the extracts were quantified by a spectrophotometer (NanoDrop-1000, Thermo, USA) and visualized by agarose gel electrophoresis.

To obtain the community-level mycobacterial *rpoB* amplicon, primers and PCR condition were referred to the ref.^25^. The 16 samples were multiplexed by the different 6-nt barcodes adding to the 5′ end of the forward or reverse primer^28^. In addition, the adaptor A and B for 454 pyrosequencing were added at the 5′-end of the barcoded forward primers and reverse primer, respectively. The PCR products were pooled at equal mass after purification. The *rpoB* amplicon pool was sent to Genome Research Center in the University of Hong Kong to perform 454 pyrosequencing (Titanium platform, Roche, USA). Genomic DNA of the four mycobacterial isolates was sent to Beijing Genomic Institute (Shenzhen, China) for Illumina sequencing on the Hiseq2000 platform with inserted length of 800 bp and paired-end (PE) reading strategy.

### Construction of the database of mycobacterial *rpoB*

A mycobacterial *rpo*B database was constructed by downloading sequences from GenBank in NCBI. The accession numbers of the *rpoB* references used in this study could be found in Table S2. All reference nucleotide sequences were trimmed to 283 bp within the primer-free amplicon region. At the time point of analysis, about 180 validated mycobacterial species were available (based on the website of *List of prokaryotic names with standing in nomenclature*). The database was constructed by *rpoB* sequences from 157 species, whose *rpoB* sequences fully covering the selected region were available. Only four of the 157 references are not from type strains, indicating the excellent taxonomic representation of the database. No sequences from subspecies were included.

### Analysis on the *rpoB* sequences

The raw pyrosequencing reads of the *rpoB* amplicons were de-multiplexed by the barcodes and trimmed by both forward and reverse primers on Mothur platform, which was originally invented for 16S rRNA sequences^29^. Quality control of the reads were performed by requiring (1) average q-value over 30 within any 20 nt windows; (2) read length over 280 nt and (3) no mismatch in the barcode and both primers. All the sequences were then performed BLASTn search against the constructed *rpoB* database. The best-hit outputs satisfying both the similarity over 93% (the cutoff was determined by an evaluation as shown in Fig. S1) and alignment length over 280 nt were kept for further analysis and the other sequences were discarded. According to the evaluation on the inter-species divergence of *rpoB*, similarity of 95% were set as the criterion for identification (we performed all-against-all *rpoB* sequence BLAST and found only about 9% inter-species pairs were higher than the cutoff of 95%). The hit was defined as the closest reference species related organism, otherwise the sequences were classified as ‘unknown *Mycobacterium*’. Simultaneously, the *rpoB* sequences across all samples were clustered into operational taxonomic units (OTUs) in the Mothur platform (using the aligned sequences of themselves as the reference for alignment), in order to calculate the taxonomy-independent mycobacterial diversity in each sample.

### Data processing for the genomic and metatranscriptomic data

The four draft genomes have been deposited in Whole Genome Shotgun Submissions in Genbank (Accession Number: *JMHQ00000000*, *JMHR00000000*, *JMHS00000000* and *JMHT00000000*). Mycobacterial gene expression in full-scale AS was investigated by revisiting a metatranscritpomic dataset^30^. The details of the genomic and metatranscriptomic analysis can be found in the section of Supplementary Methods in the Supplementary Materials.

### Experiments validating biodegradation of cholesterol by mycobacteria

The preparation of the medium with that use cholesterol as single carbon source was prepared according referred to ref.^31^. The calculated final concentration of cholesterol was 1 mM (in 50 mL Medium 457 of the DSMZ) and the cholesterol was mostly in insoluble form with homogenous dispersion in medium. The cultivation is under aerobic condition at 30 °C in a shaker (200 rpm). Because the mycobacterial isolates usually grow in cell clusters, we determined the biomass in 0, 1, 3, 7, 10 and 14 d by referring to the DNA content per mL, which were measured by using the Qubit device (High Sensitive kits, Invitrogen, USA) after bead-beating lysed cells (FastDNA SPIN Kit for Soil, MP BIOMEDICALS, USA). The cholesterol concentration during cultivation was determined by referring to reference^31^.

An activated sludge sample and the corresponding influent were collected from Qianpu WWTP (Xiamen, China) for the cholesterol enrichment assay. The influent was sterilized by filtering through 0.1 μm pore-size membrane. AS was inoculated by 1:9 (v:v) into the influent with adding 0.5 g L^−1^ cholesterol (final volume 50 mL) and cultured by shaking at 200 rpm and 25 °C with a re-inoculation at Day 7. The enrichment sludge samples were collected at 0, 7 and 14 d and the DNA was extracted by using the FastDNA Spin Kit for Soil. The total bacterial and mycobacterial rRNA gene copies were determined by absolute quantitative PCR using primer sets for 16S rRNA gene (primer set targeting V4 region^32^), and *Mycobacterium* (using primer set of MycoARB210 and MycoARB585^33^), respectively. The full-length 16S rRNA gene of SWH-M1 was ligated into the PMD-19T vector (Takara, Dalian, China), which was used to create the standard curve for the absolute qPCR of total bacterial or mycobacterial 16S rRNA genes.

## Results

### Diversity and taxonomy of mycobacteria in WWTPs

As listed in Table 1, although only a small portion of reads were filtrated out during the read quality-control step, a large number of sequences (from 36.6% to 98.7%) were removed due to their low similarity (<93%) with any mycobacterial *rpoB* reference. By searching against the online Genbank NT database, the removed sequences could be *rpoB* from diverse bacterial lineages, especially from other *Actinobacteria*, indicating the non-specific PCR amplification using the applied primer set for the complex environmental samples like AS. Finally, for each sample, from 138 to 5,715 mycobacterial *rpoB* sequences were obtained for the subsequent analysis.

**Table 1:** Profile of quality control, mycobacterial assignment and OTU classification for the raw sequences.

Sample	No. of raw reads	Reads after quality filtration	Reads assigned as mycobacterial *rpo*B^a^	No. of abundant OTUs at 98%^b^	No. of abundant OTUs at 95%^b^
US-CO	8,993	8,528	5,407	14	8
US-PC	11,151	10,548	301	13	16
CA-GU	7,293	6,926	138	21	19
EN-DE	11,448	10,738	596	11	11
SG-UP	9,827	9,109	485	12	13
CN-BJ1	7,271	6,852	167	13	17
CN-NJ	11,384	10,399	392	20	20
CN-MP	8,831	8,454	1,905	12	12
CN-ARCN	23,182	4,310	553	14	14
CN-HK-ST	11,023	10,382	779	12	12
CN-HK-SWH	10,505	10,217	603	16	13
ST-Inf	5,884	5,838	284	25	23
ST–Eff	10,789	10,606	5,715	9	10
SWH-Inf	11,000	10,552	276	17	22
SWH-Eff	10,435	9,837	868	16	15
CN-BJ2	8,924	8,499	319	14	17

^a^Sequences were over 93% similarity to a reference *rpoB* sequence.

^b^Only OTUs with abundance over 1% of total *Mycobacterium* were counted.

**Figure 1:**
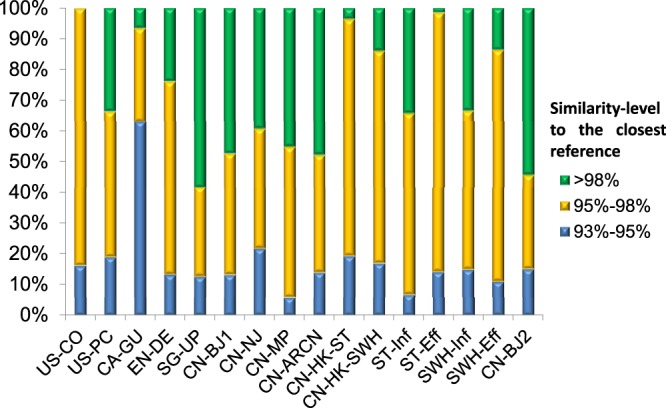
Mycobacterial *rpo*B hits at various similarity ranges in the 16 samples from 11 WWTPs.

Table 1 also showed the number of major OTUs (over 1% in all mycobacterial *rpoB* sequences in the certain sample) in each sample, which indicated the mycobacterial diversity. Nine to twenty-five OTUs at 98% similarity cutoff and eight to twenty-three OTUs at 95% similarity clustering were detected, respectively. These results indicated that diverse mycobacterial species were present in each WWTP sample, which supported previous studies that various mycobacteria could be isolated or culture-independently detected by their 16S rRNA gene in one environmental sample^34,35^. For the *rpoB* sequences assigned as *Mycobacterium*, those best-hit with similarity ranging from 93% to 95% against any reference were determined as ‘unclassified *Mycobacterium*’. There were from 5.8% to 63.0% sequences (17.4% in average) belonging to this catalog (Fig. 1). Apparently, it suggested that a significant proportion of mycobacteria, presented in the WWTPs, were distantly related to those database-covered species.

**Figure 2:**
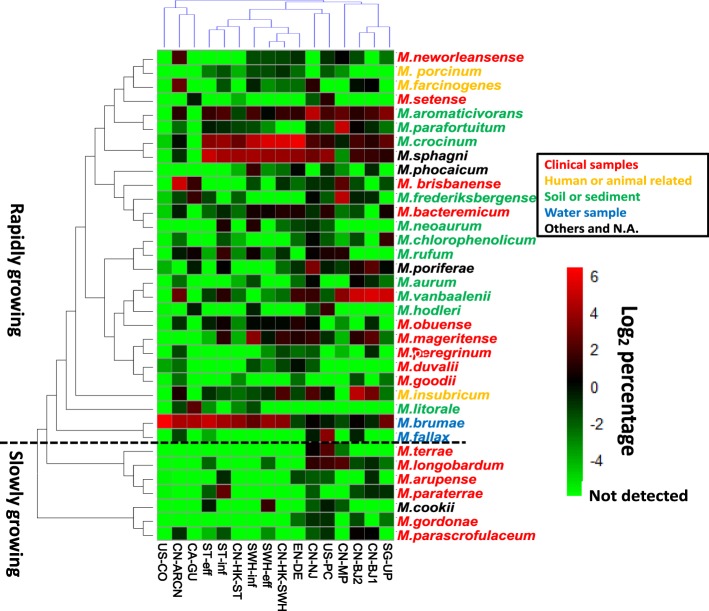
Heat-map of the relative abundance of 35 mycobacterial species in 16 samples. The 35 species were those over 2% to total *Mycobacterium* in at least one sample. Color of the reference species was based on their origin of isolation. The consensus phylogenetic tree on the left side was constructed based on the 16S rRNA gene sequences of type strains using the neighbor-join algorithm. Clustering between samples was according to their Bray-Curtis distances.

Figure 2 showed the distribution of classified mycobacterial species (>95% similarity to one reference) in all samples. Only hit species that occurred over 2% in at least one sample were displayed. For the 35 species, both slowly growing and rapidly growing groups were detected. Referring to the average relative abundance, the top five species are *M. brumae*, *M. crocinum*, *M. sphagni*, *M. vanbaalenii* and *M. aromaticivorans*, all of which are rapidly growing species, originating from natural environments (except for no origination was found for *M. sphagni*). The frequently investigated *M. vanbaalenii* was frequently investigated was a multifunctional pollutant degrader^36^. However, the other species are poorly characterized so far.

The clustering analysis among samples suggested that the mycobacteria had a poor biogeographical distribution (Fig. 2). However, the different sections of the same WWTPs exhibited a similar mycobacterial patterns according to the results from the influent, AS and effluent of ST and SWH WWTPs. This result suggested that the mycobacterial population in the influent had a significant impact on the subsequent sections, i.e., AS and effluent. One possibility is that the majority of *Mycobacterium* populations in AS are input from the influent and they were not strictly selected in the aeration tanks. However, it is interesting that both effluents from ST and SWH showed similar composition of the slowly growing species (*M. longobardum*, *M. cookii* and *M. parascrofulaceum*). Thus, wastewater treatment may have special selectivity for these slowly growing species.

Beside of the major populations, potential pathogenic mycobacteria with minor abundance were further analyzed by more rigorous criterion of over 98% similarity between query and reference *rpoB* in the customized database. Although no typical slowly-growing pathogenic mycobacteria, such as *M. tuberculosis*, *M. ulcerans*, etc., were detected at the current sampling depth, we indeed found several opportunistic pathogen species, such as *M. bacteremicum*, *M. brisbanense*, *M. fortuitum*, *M. frederiksbergense, M. neoaurum*, etc. in at least three of the 15 samples (Table 2). Therein, *M. brisbanense* and *M. frederiksbergense* were detected in six and five samples, respectively, indicating relatively wide distribution. Although the relative abundances of the opportunistic pathogen species are usually low, considering the high absolute bacterial cell density in activated sludge, the risk is not negligible. For example, in CN-HK-ST sample mycobacteria accounted for 4.0% of total bacteria and *M. fortuitum* accounted for 0.13% of total mycobacteria (Table S1 and Table 2). This means that *M. fortuitum* was about 0.0052% of total bacteria and there were 5.2 × 10^5^ cells in the one milliliter of activated sludge (given 10^10^ bacterial cells per mL). Moreover, it is still hard to conclude that the detected species have direct pathogenic risks since they may be non-pathogenic strains. One phenomenon is that *M. brisbanense* was very high relative abundance in an industrial WWTP (31.2% in CN-ARCN) without municipal sewage composition. It is less likely that the detected sequences are derived from pathogenic strains.

**Table 2:** Opportunistic mycobacterial pathogens detected in wastewater treatment plants^a^.

	Percentage to total mycobacteria %							
	*M. aubagnense*	*M. bacteremicum*	*M. brisbanense*	*M. fortuitum*	*M. frederiksbergense*	*M. hodleri*	*M. neoaurum*	*M. peregrinum*
US-CO	N.D	N.D	N.D	N.D	0.02	0.02	N.D	N.D
US-PC	N.D	N.D	0.33	N.D	N.D	1.99	0.33	N.D
CA-GU	N.D	N.D	2.17	N.D	2.90	0.72	N.D	N.D
EN-DE	N.D	0.50	0.17	N.D	N.D	N.D	0.34	0.17
SG-UP	0.62	0.62	N.D	N.D	N.D	N.D	N.D	N.D
CN-BJ	N.D	N.D	N.D	N.D	N.D	N.D	N.D	0.60
CN-NJ	N.D	N.D	0.51	N.D	0.77	N.D	0.51	N.D
CN-MP	N.D	N.D	3.31	N.D	0.89	N.D	N.D	N.D
CN-ARCN	0.18	N.D	31.28	N.D	0.18	N.D	N.D	N.D
CN-HK-ST	N.D	N.D	N.D	0.13	N.D	N.D	N.D	N.D
CN-HK-SWH	N.D	N.D	N.D	N.D	N.D	N.D	0.17	0.33
ST-Inf	N.D	0.35	N.D	N.D	N.D	N.D	2.11	N.D
ST–Eff	N.D	N.D	N.D	0.02	N.D	N.D	N.D	N.D
SWH-Inf	N.D	0.36	N.D	0.36	N.D	N.D	1.81	N.D
SWH-Eff	N.D	0.12	N.D	N.D	N.D	N.D	N.D	N.D

^a^The list of mycobacterial pathogens was referred to two documents (Brown-Elliott *et al*., 2010; Rosenblueth *et al*., 2011). Only hits with over 98% similarity to a pathogenic mycobacterial *rpo*B reference were counted and only those pathogens presented in at least two samples were listed here. The percentage is the relative abundance of the species to the total mycobacterial population.

### Mycobacterial isolates and their genomic information

The phylogenetic tree based on the full length of 16S rRNA sequences of the six isolates and reference strains is shown in Fig. S2. Isolates from the SWH WWTP showed over 99.5% similarity to references of *M. obuense* (one isolate), *M. mageritense* (three isolates) and *M. goodii* (one isolate), respectively. Noticeably, high relative abundance of *M. mageritense* has been detected in the SWH WWTP by *rpoB* profiling (16.4% of all detected mycobacteria in the influent, Fig. 2). The 16S rRNA of isolate ST-F2 from ST WWTP showed 98.9% similarity to its two closest relatives, *M. mucogenicum* and *M. phocaicum*. By referring to their *rpoB* sequences extracted from the genomes, SWH-M1, SWH-M3 and SWH-M5 were over 99% similar with the references (full length or PCR-targeted region of *rpoB* according to availability), whereas the *rpoB* of ST-F2 only exhibited 97.5% similarity with AY147171 from *M. mucogenicum* ATCC49649, suggesting its unknown taxonomic position.

The general information of the four draft genomes is listed in Table S3. Environmental mycobacteria are of interest in their biodegradation capacities on diverse pollutants^37^. As listed in Table 3, a wide spectrum of genes encoding enzymes degrading alkanes, sterols, benzoate, biphenyl, phthalate and other aromatics seemed commonly presented. A morpholine degradation operon composed of six genes as reported in *Mycobacterium* sp. RP1^38^ was also detected in SWH-M3 isolate in the exact arrangement with a high similarity of amino acid sequences (74.2–88.7%), suggesting the potential morpholine degradation capability of this strain.

**Table 3:** Biodegradation genes detected in the four draft genomes.

Enzyme	ST-F2	SWH-M1	SWH-M3	SWH-M5
Alkane 1-monooxygenase	3	2	3	1
Benzoate 1,2-dioxygenase	ND	1	1	1
3-phenylpropionate dioxygenase	1	1	1	1
Biphenyl 2,3-dioxygenase	ND	ND	1	ND
Phthalate 3,4-dioxygenase	ND	ND	1	ND
3-ketosteroid-9-alpha-hydroxylase	4	1	2	1
steroid C27-monooxygenase	1	4	1	1
Cytochrome P450 monooxygenase	34	32	47	39
*morA* operon^a^	ND	ND	1	ND

^a^The operon was referred to *Mycobacterium* sp. RP1 (Sielaff and Andreesen^38^).

**Figure 3:**
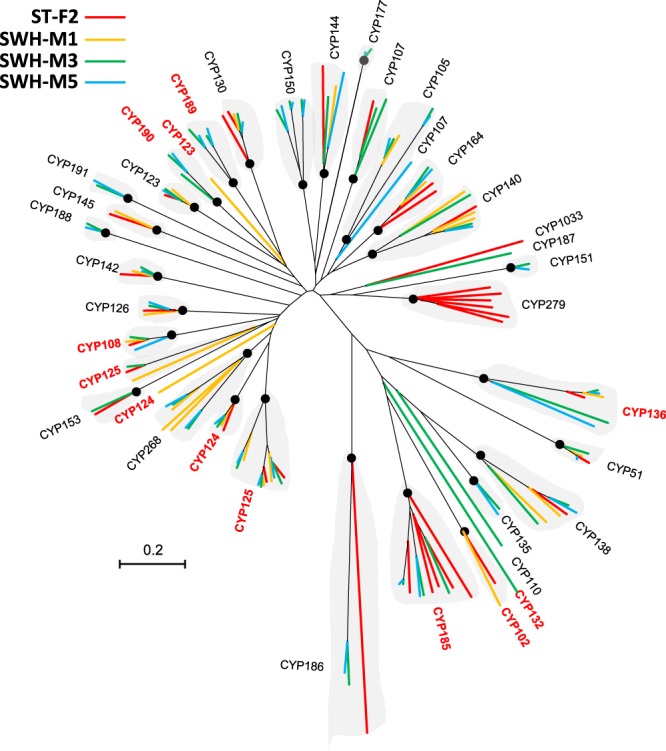
Phylogenetic tree of the 35 cytochrome P450 families (>200 aa) detected in the four mycobacterial genomes. The tree was constructed in MEGA 5 using the neighbor-joining statistical method based on Jukes-Cantor model and tested for 1,000 times of bootstrap. Black node showed that the bootstrapping confidence level for the corresponding P450 family were over 80%. Typically the amino acid sequences affiliated with such a node belong to a certain P450 family with few exceptions such as the polyphyletic phenomena in CYP107, CYP124 and CYP125. The reddish CYP family names indicated that the P450 family gene from *Mycobacterium* was detected in the metatranscriptomic dataset.

Organisms affiliated with *Actinobacteria* typically harbor multiple cytochrome P450 gene families that are monooxygenases targeting on a great number of organic compounds, especially the persistent organic pollutants^39^. As expected, we detected a large number of P450 genes and families in each genome (Table 3 and Fig. 3) and the no. of P450 gene was apparently related to the genomic size. In the 33 families exhibited in Fig. 3, fourteen were found in all the four genomes, while only five were exclusively found in a unique isolate. This result implied a significantly shared degradation spectrum among the isolates. Although the majority of detected P450 families were not well studied, families related to sterol biosynthesis (CYP51^40^,), degradation of sterols (CYP125^41^,) and alkanes (CYP153^42^,) and oxidation of methyl-branched lipids (CYP124^43^,) were determined in all the isolates. A P450 for degradation of polycyclic aromatic hydrocarbons (CYP102^44^,) was only detected in ST-F2 and SWH-M1. We also searched the genomes against a recently discovered catabolic pathway of C-19 steroids degradation containing seven critical genes, i.e., *kstD2*, *kstD3*, *kshA2*, *kshB2*, *hsaA2*, *hsaC2* and *hsaD2*^45^. If requiring the similarity over 50%, ST-F2 contains all the seven genes, while the other three genomes have only partial genes (3–4 genes).

### Metatranscriptomics hinted mycobacterial function

The above DNA-level information can only answer the potential mycobacterial functions, while information at mRNA-level can be more close to their real activities. Mycobacterial gene expression in the full-scale municipal WWTPs was profiled by revisiting the first published metatranscriptomic dataset of AS^30^. The identified mycobacterial mRNA reads were functionally assigned to the hierarchic KEGG metabolic pathways (Fig. S3). Totally, there were 509 mycobacterial mRNA reads detected and 206 of them were assigned into the metabolism catalog. In addition to the central pathways such as carbohydrate, lipid and amino acid metabolisms, other pathways of metabolisms of terpenoids, polyketides, xenobiotics and biosynthesis of secondary metabolites, were annotated at enzymatic function level. Specially, genes exclusively functioning in terpenoids biosynthesis, geraniol and sterol degradation were expressed during sampling. Therein, fifteen mRNA reads classified as the P450 families of CYP102, 108, 123, 124, 125, 132, 136, 185, 189 and 190 were also detected based on the online annotation of cytochrome P450 homepage and NR database validation (Fig. 3). Among them, CYP125 had been reported as cholesterol degrading enzyme in *Rhodococcus jostii* RHA1 and *M. tuberculosis*^41,46^.

### Experimental validation of cholesterol degradation

**Figure 4:**
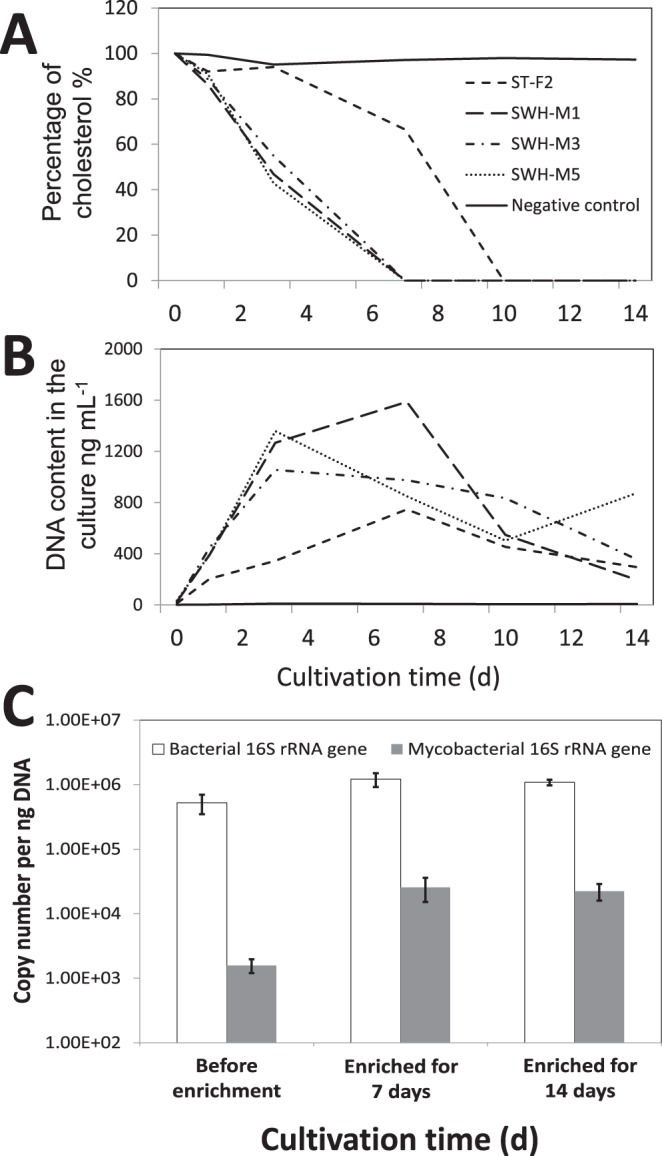
Cultivation and enrichment of *Mycobacterium* by the biodegradation of cholesterol. (**A**) Cholesterol utilization by the four isolates as single carbon source. (**B**) DNA content as the indicator of biomass growth of the four isolates during cultivation with cholesterol as single carbon source. (**C**) Enrichment of mycobacteria in the activated sludge feeding on municipal influents supplemented with 1 g L^−1^ cholesterol. Negative control in A is to use medium with cholesterol without inoculating any bacteria. Negative control in (**B**) is to grow strain ST-F2 in the medium without adding cholesterol (free of carbon source).

The genomic and metatranscriptomic results suggested that one of mycobacterial functions may be degradation of steroids, which are typically abundant in municipal sewage. Experimental results indicated that the single carbon source of cholesterol could be degraded by all isolates with the increase of their biomass (Fig. 4A,B), confirming that at least some mycobacteria in AS could degrade the typical sterols in sewage. On the other hand, after enrichment by adding cholesterol into the filtrated influent, the relative abundance the 16S rRNA gene copies of mycobacteria to total bacteria in the AS increased from 0.85% to 4.04% and 3.11% (4.7 and 3.7 fold) on the seventh day and the fourteenth day, respectively (Fig. 4C). Together with the metatranscriptomic results, these findings validated that *Mycobacterium* in AS play a significant role in degradation of the cholesterol in the municipal sewage.

## Discussion

*Mycobacterium* are moderately abundant genus that can be up to 4% of total bacteria^7^. It was thought to be foaming bacteria causing adverse operational problems during sewage treatment^5,47^. For the Shatin WWTP, our previous study also found that *Mycobacterium* were not significantly enriched in the foam compared with the AS phase^48^. According to a five-year monthly monitoring of the bacterial community in Shatin WWTPs, abundances of *Mycobacterium* were quite stable^49^. By using the five-year monitoring data, the mycobacterial abundances in AS related with the operational parameters were analyzed. As shown in Fig. S4 (canonical correspondence analysis for the variation of total mycobacteria and 6 subgroups and parameters), the abundance of total mycobacteria hardly interacted with any presented physiochemical factors in the influent. Such observation, consistent with the high frequency of *Mycobacterium* in global WWTPs^7^, indicated that *Mycobacterium* stably occupied certain nutritional niche in the WWTPs. However, Fig. S4 also showed that factors such as BOD, temperature, SRT, etc., could affect the variation of different subgroups, which may include those potential pathogenic *Mycobacterium* species as detected in this study. Noticeably, Species of atypical *Mycobacterium* had shown resistance to chlorination^50^, which is a common processing to minimizing hygienic impacts of AS treatment. In order to reduce pathogenic risk, as suggested by our results that multiple opportunistic mycobacterial pathogens can be detected in WWTPs, it is important to understand the pathogenicity of the strains and the effect of operational parameters on those pathogenic mycobacterial species in the future studies.

As the first detailed investigation on mycobacterial diversity and functions in AS for sewage treatment, we found that nearly all abundant mycobacterial populations identified in the present study, such as *M*. *brumae*, *M*. *crocinum*, *M. sphagni*, *M. aromaticivorans* and the isolates, were species without extensive studies on their eco-physiology, which raises further interests to study their physiology in wastewater treatment. Interestingly, three recently established species isolated from Hawaiian soils, i.e., *M. crocinum*, *M. aromaticivorans*, *M. rufum*, co-occurred on the list of species^51^, suggesting that some *Mycobacterium* species in WWTPs may be indigenous species of soil.

So far, a great number of cytochrome P450 family genes and PAHs degradation capabilities have been found in environmental *Mycobacterium* isolates^52,53^. However, very few species were isolated from the municipal or industrial wastewater treatment plants. Our investigation indicated that mycobacteria populations in AS are highly diverse and intriguingly novel according to their *rpoB* sequences. More efforts should be made to obtain more isolates from the wastewater treatment systems. Meanwhile, the opportunistic mycobacterial pathogens detected suggested the possible health risk of mycobacteria in WWTPs although the chance may not be high as showed by our results. It raised concerns to further study the viability of these organisms (i.e., live or dead) in effluent and the efficiency of disinfection methods to remove them.

A report has shown that most *Mycobacterium* cells in the influent were removed after decantation, implying that these organisms linked with large particles as determined by their hydrophobic nature^54^. Sterols, mostly cholesterol, are the significant sewage components that are relatively resistant to biological degradation^55^ Their high apolarity determines their low solubility and association with large particles, perhaps the other insoluble fecal materials. Thus, our results also support that at least some mycobacteria in AS are particle-associated and related to the influent populations (Fig. 1B). Moreover, it is more important that the metatranscriptomic data and the test of cell growth on cholesterol indicated that mycobacteria played a role in sterols degradation in WWTPs. Thus, for the first time, this study proposes the functional roles of *Mycobacterium* as degraders of sterols in biological treatment of municipal wastewater. Furthermore, we also confirmed that all the four isolates could grow readily on filtrated particle-free sewage influent (data not shown), indicating that they can easily degrade soluble organics in sewage. Therefore, besides the function of sterol degradation proposed in the present study, mycobacterial species may also have roles in biodegradation of other pollutant.

In summary, the study presents the first detailed investigation on mycobacterial diversity and functions in the activated sludge for sewage treatment. Nearly all abundant mycobacterial populations were so far poorly characterized species such as *M. brumae*, *M. crocinum*, *M. sphagni*, etc. Mycobacterial function of sterol degradation that was proposed by genomic and metatranscriptomic data, as well as the experimental validation of isolates. Meanwhile, although a few opportunistic pathogens like *M. brisbanense* and *M. frederiksbergense* were detected, a comprehensive study based on more AS samples will provide more reliable information about the existence and distribution of pathogenic mycobacteria in wastewater treatment systems.

## Supplementary information

SUPPLEMENTARY INFO
