# Supplementary material for: Mycobacterial species and their contribution to cholesterol degradation in wastewater treatment plants
Source: Sci Rep. 2019 Jan 29;9:836. doi: 10.1038/s41598-018-37332-w (PMC6351609; doi:10.1038/s41598-018-37332-w)
Supplement: Supplementary file 1 — SUPPLEMENTARY INFO [file 41598_2018_37332_MOESM1_ESM.pdf]

Supplementary materials for ***Scientific Reports***

Mycobacterial species and their contribution to cholesterol degradation in wastewater treatment plants

Feng Guo[1*], Tong Zhang[2], Bing Li[3], Zhiping Wang[4], Feng Ju[2], Yi-ting Liang[1]

1. School of Life Sciences, Xiamen University, Xiamen, China
2. Environmental Biotechnology Laboratory, The University of Hong Kong, Hong Kong SAR, China
3. Key Laboratory of Microorganism Application and Risk Control of Shenzhen, Graduate School at Shenzhen, Tsinghua University, China
4. School of Environmental Science and Engineering, Shanghai Jiao Tong University

*Correspondence author

Email: fguo.bio@xmu.edu.cn

Tel: +86-592-2880330

Fax: +86-592-2880330

Supplementary Methods

Data processing for the genomic data

The two *fastq* files (PE reads) for each genome were imported to the CLC 6.0 software and assembled using Kmer length of 51. The obtained scaffolds over 1,000 nt length were kept as the draft genome. Open reading frames (ORFs) were predicted with the online server of GeneMark (Besemer and Borodovsky, 2005). The four draft genomes have been deposited in Whole Genome Shotgun Submissions in Genbank (Accession Number: *JMHQ00000000*, *JMHR00000000*, *JMHS00000000* and *JMHT00000000*).

Determination of the biodegradation genes in the genomes

To determine the biodegradation genes in the four mycobacterial genomes, the FunGene database was referred (Fish et al., 2013). The genomes (encoded protein) were performed BLASTP against the database and only hits with over 100 bit-score and 50% similarity to a reference were kept. Then the hit sequences were conducted BLASTP against NR database and manually checked the annotations of their relatives to avoid false-positive results. Especially, the cytochrome P450 monooxygenases in the genomes were initially identified by Pfam (Punta et al., 2012) and further classified into families on the online BLAST server of homepage of cytochrome P450 (http://blast.uthsc.edu/) using the bacterial references (Nelson, 2009). The cytochrome P450 ORFs (completed amino acid sequences) in the draft genomes were analyzed by constructing a consensus neighbor-joining phylogenetic tree using MEGA 5 software (Tamura et al., 2011).

Mycobacterial gene expression based on metatranscriptomic data

A metatranscriptomic dataset (about 1.4 million Illumina PE reads with the length of 90 nt) derived from another WWTP in Hong Kong was revisited to profile mycobacterial gene expression during full-scale sewage treatment (Yu and Zhang, 2012). To achieve this, first we conducted the BLASTn of the metatranscriptomic dataset against the combined database of the four draft genomes containing the protein-coding genes. Only reads with over 25 aa aligned length and 80% similarity were marked as candidate mycobacterial mRNA reads. Then the candidates were further filtered by performing BLASTx (filtered by e-value of 10^{-5} and output 10 hits) against the Non-redundant protein database and those taxonomically annotated as *Mycobacterium* by MEGAN (Huson et al., 2007) under the default lowest common ancestor settings were further extracted as confident mycobacterial mRNA reads. The reads were annotated in MEGAN under the KEGG system (Kanehisa et al., 2010).

Tables

Table S1 Summary of sampled wastewater treatment plants

ID	Abbreviation	Location	Process[a]	Percentage (%) of sewage	COD mg L^{-1}	TN mg L^{-1}	Sampling time	Temperature at sampling ^{o}C	Relative abundance of *Mycobacterium* %[b]
01	US-CO	Columbia Regional (Columbia, USA)	CAS	76	700	32	04/2010	17	0.53
02	US-PC	Potato Creek (Griffin, USA)	OD	Predominant	402	30	05/2010	18	0.61
03	CA-GU	Guelph (Guelph, Canada)	CAS	55	270	45	01/2010	13	0.18
04	EN-DE	Derby (Derby, England)	N.A.	N.A.	N.A.	N.A.	11/2012	N.A.	N.A.
05	SG-UP	Ulu Pandan (Singapore)	CAS+MBR	Predominant	265	45	05/2010	27	0.55
06	CN-BJ1	Bei-Xiao-He (Beijing, PRC)	A/A/O+ MBR	95	462	51	12/2009	16	0.81
07	CN-NJ	Suo-Jin-Cun (Nanjing, PRC)	A/O	85	330	57	11/2009	22	0.28
08	CN-MP	Shanghai, PRC	A/O	0	6,000	246	08/2012	25	3.55
09	CN-ARCN	Shanghai, PRC	A/O	0	2,000	197	08/2012	25	0.25
10	CN-HK-ST	Shatin (Hong Kong, PRC)	A/O	95	329	31	05/2013	23	4.00
11	CN-HK-SWH	Shekwuhui (Hong Kong, PRC)	A/O	90	343	30	05/2013	23	1.06
12	ST-Influent	Shatin (Hong Kong, PRC)	/	/	/	/	05/2013	23	7.12
13	ST--Effluent	Shatin (Hong Kong, PRC)	/	/	<40	<3	05/2013	23	0.65
14	SWH-Influent	Shekwuhui (Hong Kong, PRC)	/	/	/	/	05/2013	23	N.A.
15	SWH-Effluent	Shekwuhui (Hong Kong, PRC)	/	/	<40	<3	05/2013	23	N.A.
16	CN-BJ2	Bei-Xiao-He (Beijing, PRC)	A/A/O+ MBR	95	462	51	12/2009	16	0.81

[a] All activated sludge samples were collected from the aeration tanks.

[b] The relative abundance of 13 samples were retrieved from three studies (Ju et al., 2014; Ye and Zhang, 2013; Zhang et al., 2012;)

Table S2 Organisms and sequences involved in construction of the mycobacterial *rpo*B databases

Accession No.	Species	Accession No.	Species
CU458896.1	*M. abscessus*	EU191925.1	*M. kumamotonense*
AF057450.1	*M. africanum*	AB370178.1	*M. kyorinense*
AY544881.1	*M. agri*	AY544938.1	*M. lacus*
EF472961.1	*M. aichiense*	AY544939.1	*M. lentiflavum*
HG673740.1	*M. algericum*	AF057474.1	*M. leprae**
AY544883.1	*M. alvei*	HG673737.1	*M. litorale*
KC010489.1	*M. arabiense*	LQPG01000001.1	*M. longobardum*
DQ841183.1	*M. aromaticivorans*	AY544940.1	*M. madagascariense*
HQ287516.1	*M. arosiense*	AY544941.1	*M. mageritense*
MVHH01000051.1	*M. arupense*	AF057475.1	*M. malmoense*
AF057455.1	*M. asiaticum*	FJ232524.1	*M. mantenii*
DQ987716.1	*M. aubagnense*	AF057476.1	*M. marinum*
AF057456.2	*M. aurum*	HQ287518.1	*M. marseillense*
AY544886.1	*M. austroafricanum*	AY544944.1	*M. microti*
AY544889.1	*M. avium*	MVHZ01000001.1	*M. minnesotense*
MVHJ01000002.1	*M. bacteremicum*	EU191923.1	*M. monacense*
FUWC01000001.1	*M. boenickei*	AY943193.1	*M. montefiorense*
AY544890.1	*M. bohemicum*	AY544945.1	*M. moriokaense*
AY859692.1	*M. bolletii*	AY147170.1	*M. mucogenicum*
AY544891.1	*M. botniense*	AY544947.1	*M. murale*
MVHL01000001.1	*M. bouchedurhonense*	DQ124108.1	*M. nebraskense*
HF566387.1	*M. bourgelatii* *	AF057477.1	*M. neoaurum*
AF057451.1	*M. bovis*	AY943188.1	*M. neworleansense*
AY544895.1	*M. branderi*	AF057478.1	*M. nonchromogenicum*
AY943185.1	*M. brisbanense* *	EU810775.1	*M. noviomagense*
AY544896.1	*M. brumae*	AY544950.1	*M. novocastrense*
AY943186.1	*M. canariasense*	AY544951.1	*M. obuense*
AY544972.1	*M. caprae*	DQ534001.1	*M. pallens*
AF057458.1	*M. celatum*	LQPJ01000147.1	*M. palustre*
AY147163.1	*M. chelonae*	LQPM01000001.1	*M. paraense*
AY943187.1	*M. chimaera*	EF472959.1	*M. parafortuitum*
AF057461.1	*M. chitae*	KC525206.1	*M. paragordonae*
AY544900.1	*M. chlorophenolicum*	NCXN01000038.1	*M. paraintracellulare*
AY544901.1	*M. chubuense*	JF271825.1	*M. parakoreense*
AFVW02000001.1	*M. colombiense*	JF271829.1	*M. parascrofulaceum*
EU191922.1	*M. conceptionense*	HQ287519.1	*M. paraseoulense*
AY544902.1	*M. confluentis*	EU919230.1	*M. paraterrae*
AY544903.1	*M. conspicuum*	AY943191.1	*M. parmense*
AY544904.1	*M. cookii*	AF057481.1	*M. peregrinum*
DQ124109.1	*M. cosmeticum*	AF057480.1	*M. phlei*
DQ534002.1	*M. crocinum*	AY859693.1	*M. phocaicum*
AY544905.1	*M. diernhoferi*	AY544955.1	*M. porcinum*
AY544906.1	*M. doricum*	AY544956.1	*M. poriferae*
AY544907.1	*M. duvalii*	DQ987711.1	*M. pseudoshottsii*

AY544908.1	*M. elephantis*	AY943194.1	*M. psychrotolerans*
LQOT01000001.1	*M. engbaekii*	AY544957.1	*M. pulveris*
LQOU01000001.1	*M. europaeum*	AY544958.1	*M. rhodesiae*
AF057462.1	*M. fallax*	DQ841184.1	*M. rufum*
AY544910.1	*M. farcinogenes*	DQ534006.1	*M. rutilum*
AF057463.1	*M. flavescens*	HQ692917.1	*M. salmoniphilum*
DQ987713.1	*M. florentinum*	MVII01000002.1	*M. saopaulense*
DQ987721.1	*M. fluoranthenivorans*	AY943192.1	*M. saskatchewanense*
AF057464.1	*M. fortuitum*	AF057482.2	*M. scrofulaceum*
LQOW01000024.1	*M. fragae*	AF057483.1	*M. senegalense*
MVHQ01000008.1	*M. franklinii*	FJ268578.1	*M. senuense*
AY544914.1	*M. frederiksbergense*	EU191926.1	*M. seoulense*
AY544915.1	*M. gadium*	AY544961.1	*M. septicum*
AF057466.1	*M. gastri*	JTJW01000005.1	*M. setense*
AF057467.1	*M. genavense*	LQPT01000069.1	*M. sherrisii*
AY544917.1	*M. gilvum*	AF057486.1	*M. shimoidei*
AY544918.1	*M. goodii*	AB268504.2	*M. shinjukuense*
AF057468.1	*M. gordonae*	AF057484.2	*M. simiae*
AF057469.2	*M. haemophilum*	AF057485.1	*M. smegmatis*
AY544921.1	*M. hassiacum*	AY544965.1	*M. sphagni*
AY544922.1	*M. heckeshornense*	AF057487.1	*M. szulgai*
AY544923.1	*M. heidelbergense*	AF057488.1	*M. terrae*
LZKB01000106.1	*M. heraklionense**	AF057489.1	*M. thermoresistibile*
AY544924.1	*M. hiberniae*	AY544969.1	*M. tokaiense*
AY544925.1	*M. hodleri*	AY544970.1	*M. triplex*
AY544926.1	*M. holsaticum*	AF057490.1	*M. triviale*
LQPA01000001.1	*M. immunogenum*	AF057454.1	*M. tuberculosis*
MVHS01000036.1	*M. insubricum*	AF057491.1	*M. ulcerans*
AF057470.2	*M. interjectum*	AF057492.1	*M. vaccae*
AF057471.1	*M. intermedium*	AY544977.1	*M. vanbaalenii*
AF057472.1	*M. intracellulare*	NCXM01000019.1	*M. vulneris*
AF057473.2	*M. kansasii*	AY544978.1	*M. wolinskyi*
AY544936.1	*M. komossense*	AF057493.1	*M. xenopi*
JF271828.1	*M. koreense*	CP003347.1	*M. yongonense*
AY544937.1	*M. kubicae*		

*The sequence is not from the type strain of the species.

Table S3 Summarization of the four mycobacterial draft genomes

	ST-F2	SWH-M1	SWH-M3	SWH-M5
No. of contigs or scaffolds	101	247	329	231
Genome size (M)	6.0	5.8	8.4	6.9
N50	128,684	43,616	36,055	53,240
GC content %	66.8	67.8	66.8	67.0
Coding density %	92.4	92.9	92.1	91.9
Protein-coding genes	5,659	5,674	8,471	6,831
Plasmid	ND	ND	1	ND

ND: not detected.

Figures

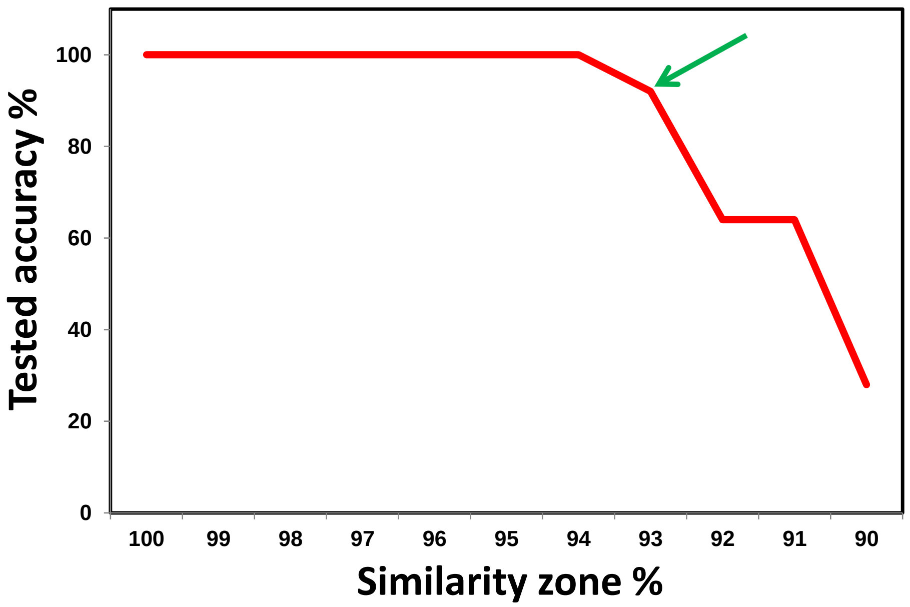

Figure S1 Test of taxonomic confidence of hits in different similarity zone between queries and mycobacterial *rpo*B reference in our database. For each similarity zone (1% scale, e.g., 98-99%), one hundred hits were randomly picked. Their sequences were performed BLASTn against online NT database. One sequence was considered to be mycobacterial *rpo*B only if the best hit was a reference of mycobacterial *rpo*B. The curve suggests that 93% is a sound cutoff for identifying mycobacterial *rpo*B sequences based on our database.

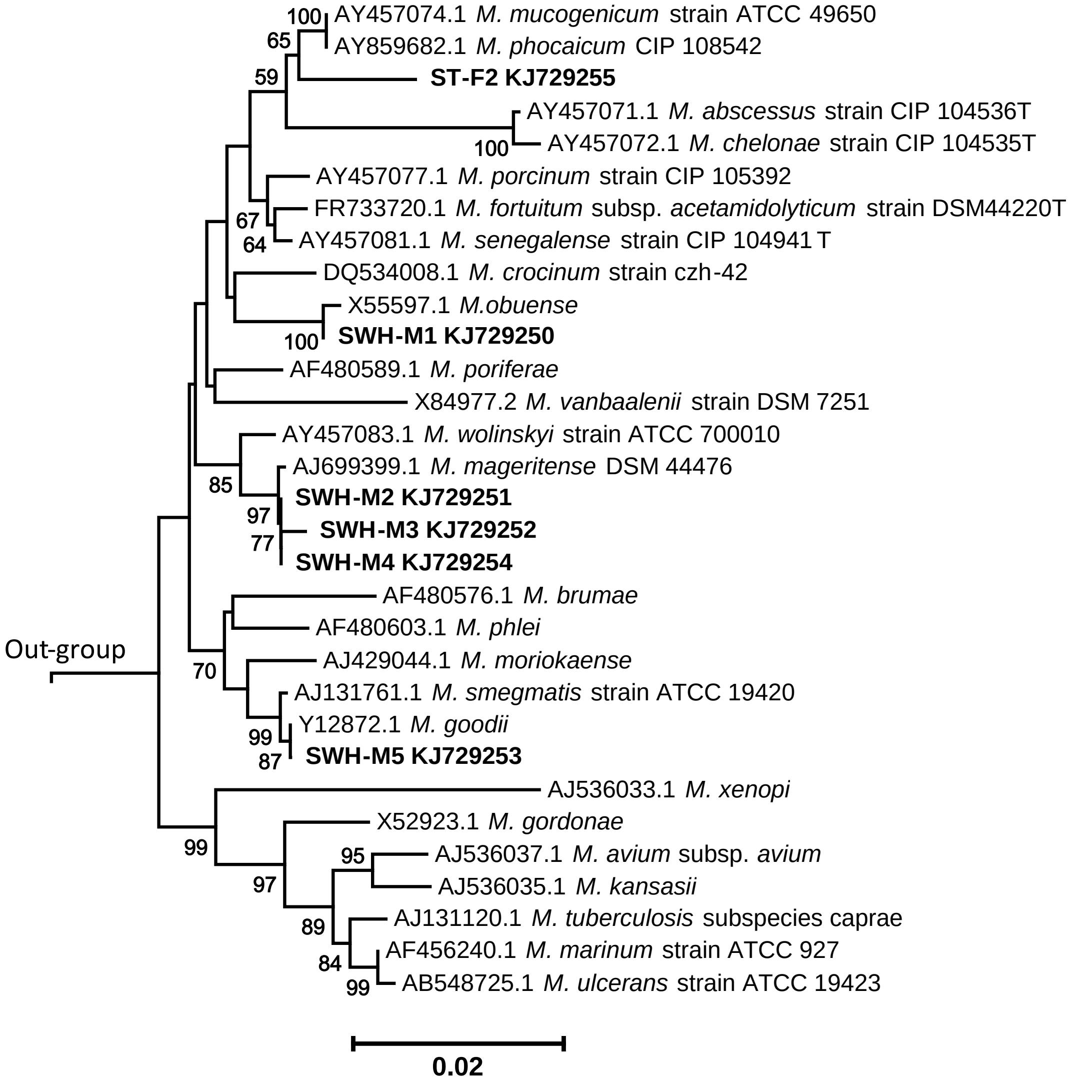

Figure S2 Phylogenetic placements of the six mycobacterial isolates referring to their full length 16S rRNA sequences. Neighbor-joining clustering method based on Jukes-Cantor model and 1000 times of bootstrap was applied during construction of the phylogenetic tree on MEGA 5 (Tamura et al., 2011). The numbers aside of the nodes showed the bootstrapping confidence level.

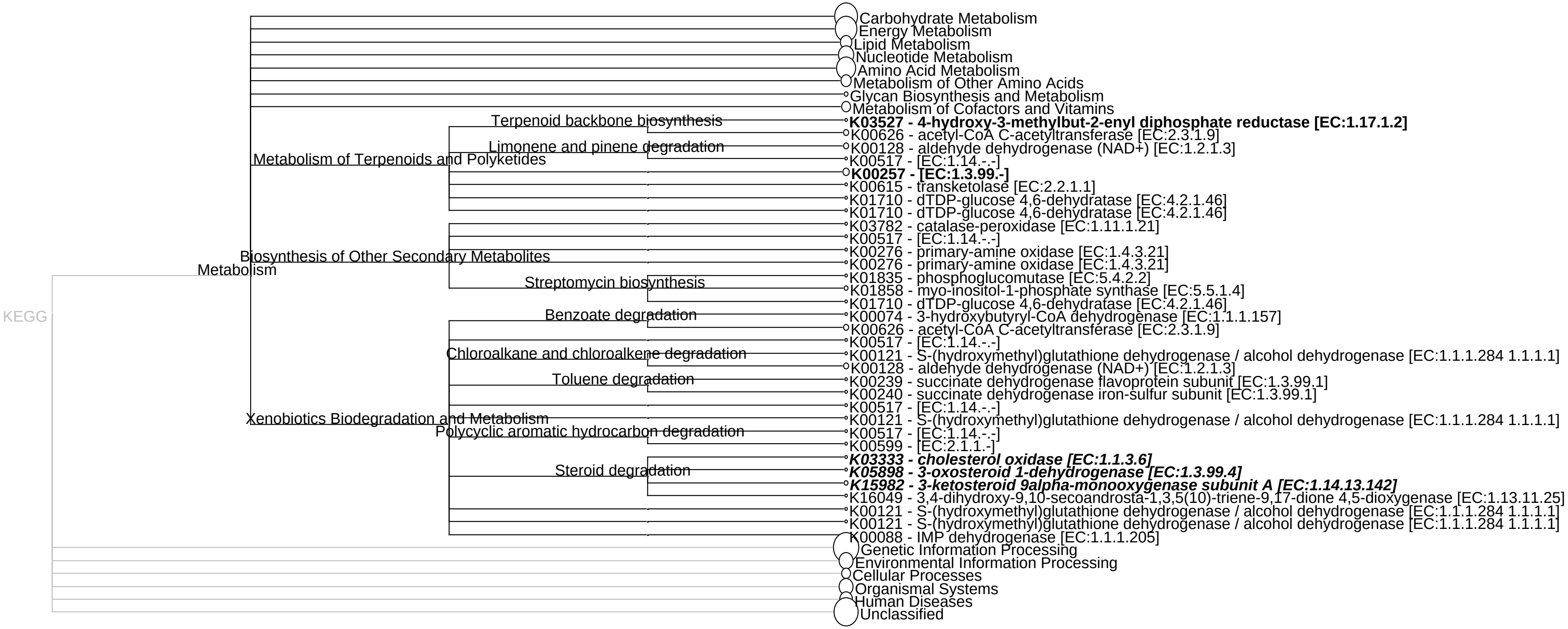

Figure S3 Mycobacterial gene expression profile in wastewater treatment plant revealed by the metatranscriptomic datasets from a full scale activated sludge. The detected mycobacterial mRNA involved in metabolism of terpenoids, polyketides, xenobiotics and biosysthesis of secondary metabolites were collapsed to functional level. The genes were formatted in italic and bold style if they were found exclusively participating in the affiliated KEGG pathways. The cycle size reflected the number of hits and the smallest ones are one hit.

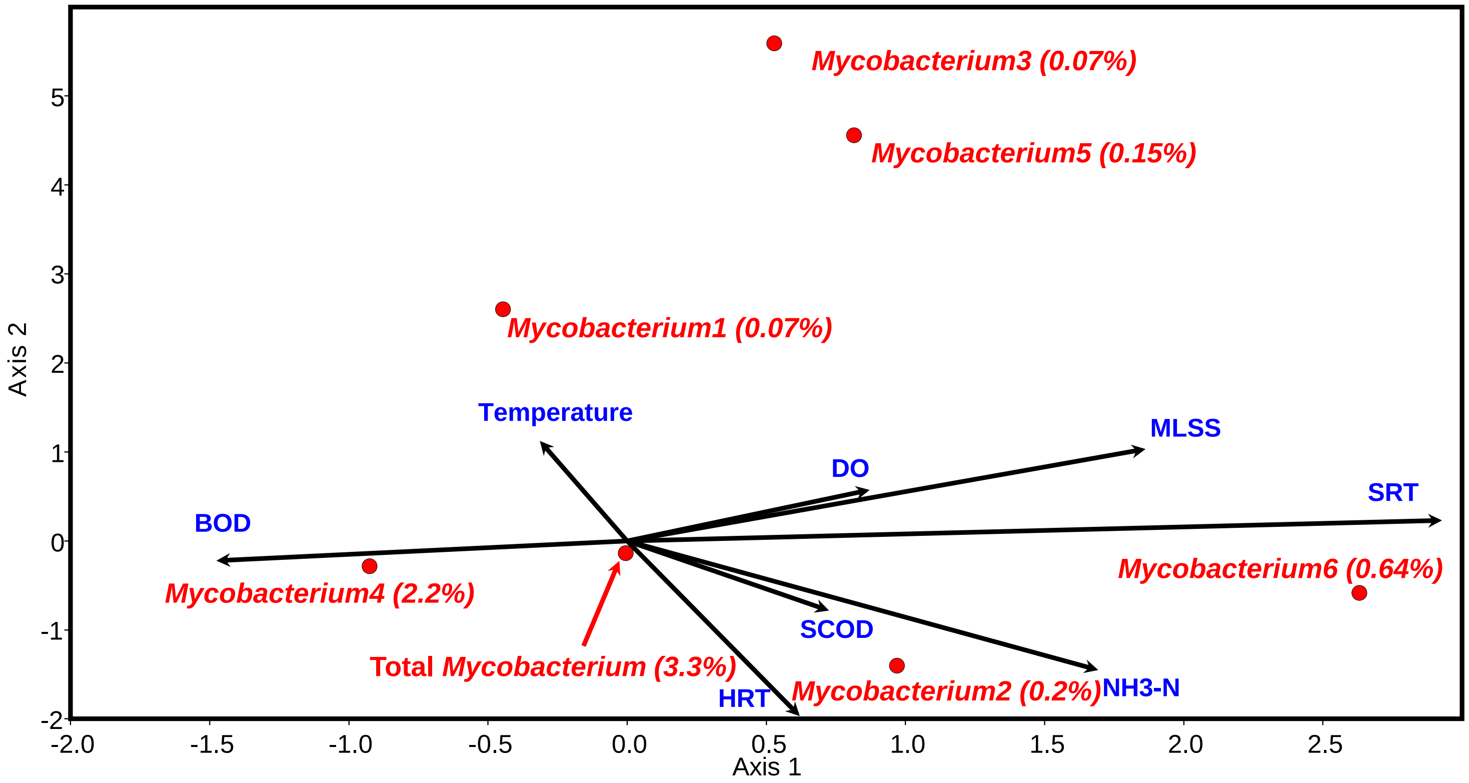

Figure S4 CCA analysis showed the variations of total *Mycobacterium* and its subgroups in Shatin WWTP with the environmental parameters (monthly average) in a monthly manner during five years. The original datasets has been published before by the authors' group [9]. There are six mycobacterial OTUs based on their 16S rRNA similarity. The percentages in the bracket are the average abundance during the five years. Total *Mycobacterium* showed stable abundance and no parameter could explain its limited variation in the WWTP.

References for the Supplementary Materials

Besemer, J., Borodovsky, M., 2005. GeneMark: web software for gene finding in prokaryotes, eukaryotes and viruses. Nucleic Acids Res. 33 (suppl 2), W451-W454.

Fish, J.A., Chai, B., Wang, Q., Sun, Y., Brown, C.T., Tiedje, J.M., Cole, J.R., 2013. FunGene: the functional gene pipeline and repository. Front. Microbiol. 4, 291.

Huson, D.H., Auch, A.F., Qi, J., Schuster, S.C., 2007. MEGAN analysis of metagenomic data. Genome Res. 17, 377-386.

Ju F, Xia Y, Guo F, et al. Taxonomic relatedness shapes bacterial assembly in activated sludge of globally distributed wastewater treatment plants. Environ Microbiol, 2014, 16(8): 2421-2432.

Ju, F. Zhang, T. 2015. Bacterial assembly and temporal dynamics in activated sludge of a full-scale municipal wastewater treatment plant. ISME J, 9(3), 683-695.

Kanehisa, M., Goto, S., Furumichi, M., Tanabe, M., Hirakawa, M., 2010. KEGG for representation and analysis of molecular networks involving diseases and drugs. Nucleic Acids Res. 2010, 38, 355-360.

Nelson, D.R., 2009. The cytochrome p450 homepage. Human Genomics 4, 59-65.

Punta, M., Coggill, P.C., Eberhardt, R.Y., Mistry, J., Tate, J., Boursnell, C., Pang, N., Forslund, K., Ceric, G., Clements, J., Heger, A., Holm, L., Sonnhammer, E.L., Eddy, S.R., Bateman, A., Finn, R.D., 2012. The Pfam protein families database. Nucleic Acids Res. 40, 290-301.

Tamura, K., Peterson, D., Peterson, N., Stecher, G., Nei, M., Kumar, S., 2011. MEGA5: molecular evolutionary genetics analysis using maximum likelihood, evolutionary distance, and maximum parsimony methods. Mol. Biol. Evol. 28, 2731-2739.

Ye L, Zhang T. Bacterial communities in different sections of a municipal wastewater treatment plant revealed by 16S rDNA 454 pyrosequencing. Appl Microbiol Biotechnol, 2013, 97(6): 2681-2690.

Yu, K., Zhang, T., 2012. Metagenomic and metatranscriptomic analysis of microbial community structure and gene expression of activated sludge. PLoS ONE 7, e38183.
